# Immature Blood Vessels in Rheumatoid Synovium Are Selectively Depleted in Response to Anti-TNF Therapy

**DOI:** 10.1371/journal.pone.0008131

**Published:** 2009-12-02

**Authors:** Elena Izquierdo, Juan D. Cañete, Raquel Celis, Begoña Santiago, Alicia Usategui, Raimon Sanmartí, Manuel J. del Rey, José L. Pablos

**Affiliations:** 1 Servicio de Reumatología, Hospital 12 de Octubre, Madrid, Spain; 2 Unitat d'Artritis, Servei de Reumatologia, Hospital Clínic de Barcelona, IDIBAPS, Barcelona, Spain; University of Pittsburgh, United States of America

## Abstract

**Background:**

Angiogenesis is considered an important factor in the pathogenesis of Rheumatoid Arthritis (RA) where it has been proposed as a therapeutic target. In other settings, active angiogenesis is characterized by pathologic, immature vessels that lack periendothelial cells. We searched for the presence of immature vessels in RA synovium and analyzed the dynamics of synovial vasculature along the course of the disease, particularly after therapeutic response to TNF antagonists.

**Methodology/Principal Findings:**

Synovial arthroscopic biopsies from RA, osteoarthritis (OA) and normal controls were analyzed by double labeling of endothelium and pericytes/smooth muscle mural cells to identify and quantify mature/immature blood vessels. To analyze clinicopathological correlations, a cross-sectional study on 82 synovial biopsies from RA patients with variable disease duration and severity was performed. A longitudinal analysis was performed in 25 patients with active disease rebiopsied after anti-TNF-α therapy. We found that most RA synovial tissues contained a significant fraction of immature blood vessels lacking periendothelial coverage, whereas they were rare in OA, and inexistent in normal synovial tissues. Immature vessels were observed from the earliest phases of the disease but their presence or density was significantly increased in patients with longer disease duration, higher activity and severity, and stronger inflammatory cell infiltration. In patients that responded to anti-TNF-α therapy, immature vessels were selectively depleted. The mature vasculature was similarly expanded in early or late disease and unchanged by therapy.

**Conclusion/Significance:**

RA synovium contains a significant fraction of neoangiogenic, immature blood vessels. Progression of the disease increases the presence and density of immature but not mature vessels and only immature vessels are depleted in response to anti-TNFα therapy. The different dynamics of the mature and immature vascular fractions has important implications for the development of anti-angiogenic interventions in RA.

## Introduction

Increased synovial vascularity and biomarkers of angiogenesis have been described in different chronic arthritic diseases [1]–[6]. Multiple inflammatory mediators such as cytokines, chemokines and growth factors produced in excess in the synovial environment can directly or indirectly mediate inflammatory angiogenesis [5]–[7]. One of the key mediators of the inflammatory angiogenic response is vascular endothelial growth factor (VEGF). VEGF can be induced by hypoxia and cytokines in synovial macrophages and fibroblasts [5]–[9]. Local and systemic levels of VEGF have been found increased in rheumatoid arthritis (RA) and correlate with active and severe disease [8]–[12]. In the collagen induced arthritis murine model, different VEGF antagonists have consistently shown remarkable therapeutic effects, pointing to angiogenesis as a valid therapeutic target [13]–[15]. However, detailed morphological studies of the changes in vascularity or vascular structure in arthritic tissues after therapy are lacking in this model. VEGF is also an important regulator of vascular permeability and participates in myeloid cell migration and function [16]–[18]. Therefore, its antagonists might also improve arthritis by down-regulating these processes, also highly relevant to the pathogenesis of arthritis

VEGF mediated pathological angiogenesis has been extensively analyzed in cancer, where VEGF antagonists have reached clinical use and benefit patients with advanced malignancies [19]. Cancer angiogenesis is characterized by morphologically abnormal, immature, dilated and leaky vessels, which decrease effective tumour perfusion and contribute to tumour development by multiple mechanisms [20], [21]. These VEGF induced immature vessels lack proper periendothelial coverage by pericytes or smooth muscle cells (SMC). VEGF mediates endothelial proliferation while inhibiting pericyte and SMC development, a process instead dependent on platelet derived growth factor (PDGF) signalling [22], [23]. Selective depletion of immature vessels has been demonstrated after VEGF targeting in animal models of cancer, whereas mature vessels are relatively stable and resistant to VEGF antagonists [20]–[24]. VEGF inhibition retards tumour progression by complex effects in vascular functions, including improved effective tumour perfusion and changes in inflammatory cell and fluid influx [22]–[25].

Similar to tumours, in RA synovium, a severely hypoxic environment is maintained despite active angiogenesis and enhanced vascularity, suggesting abnormal function of the neoangiogenic vessels [26], [27]. However, the presence of immature synovial vessels or their potential contribution to the disease process has not been investigated. Improvement of the disease in response to anti-TNF therapy is paralleled by a dramatic reduction in local or systemic VEGF and other angiogenesis markers [10]–[12], [28], [29]. Imaging techniques suggest that increased vascularity and oedema are reduced by effective therapy [30]–[32]. Persistent vascular activity correlates with further damage to bone and cartilage tissues even in patients on clinical remission. Therefore, analyzing changes in vascular structure and density after the indirect VEGF down regulation that occurs in response to anti-TNF-α therapy might be informative on the potential role of neoangiogenic vessels in the pathogenesis of RA.

We have specifically analyzed the pericyte/endothelial structure of RA synovial vessels and whether changes in vascular density or maturity correlate with clinicopathological progression of the disease. Furthermore, we longitudinally analyzed potential changes in the vascular structure in response to effective therapy in a series of patients treated with TNF-α antagonists for active disease.

## Methods

### Ethics Statement

All patients signed a written informed consent. The present study was approved by the institutional ethical committees of both participating centers (Ethical Committee of the Hospital Clinic of Barcelona, Barcelona, and Clinical Research Ethics Committee of the Hospital 12 de Octubre, Madrid, Spain).

### Patients and Synovial Biopsies

Arthroscopic synovial tissue biopsies were obtained from the knee of 82 patients fulfilling the American Rheumatism Association revised criteria for RA. All patients had active disease characterized by inflammation of at least one knee joint. Patients characteristics at biopsy, including age, sex, disease duration, 28-joint Disease Activity Score (DAS28), C-reactive protein and erythrocyte sedimentation rate (ESR), presence of IgM rheumatoid factor (RF) (positive≥30 IU/ml) or anti-citrullinated protein antibodies (ACPA) as determined by second-generation enzyme-linked immunosorbent assay (positive≥50 IU/ml, Immunoscan, Stockholm, Sweden), and the presence of erosions were recorded.

A subgroup of 25 patients that started an anti-TNF-α therapy (etanercept, adalimumab or infliximab) at first biopsy due to active disease refractory to previous DMARD therapy (mean DAS28 score 6.0±1.4), underwent a second biopsy after 10±2 months of anti-TNF-α therapy. All these patients also received DMARD therapy with methotrexate (7.5–20 mg/week) and 60% low dose prednisone (≤5 mg/day). Arthroscopic biopsies were obtained for research purposes as previously described (33). The rate of side effects of arthroscopy was very low and restricted to delayed wound healing of one of the portals of entry in one patient (<1%). After arthroscopy, lavage and steroid injection were performed and usually followed by rapid improvement of arthritic pain.

Control synovial tissues from 14 osteoarthritic (OA) synovial tissues were obtained by synovectomy at prostetic join replacement surgery. In addition, normal (non-inflammatory) synovial tissues were obtained from 4 individuals lacking previous joint disease at elective arthroscopic surgery for minor traumatic lesions. Lack of inflammatory infiltration in these tissues was confirmed by histological examination.

### Immunofluorescent Labelling of Synovial Vessels

Tissues were deparafinized, rehydrated and microwave heated in pH 9 EDTA for antigen retrieval. Double immunofluorescent labeling of endothelium and periendothelial pericytes/smooth muscle cells was performed by sequential incubation with anti-CD31 (JC70A clone, Dako, Carpinteria, CA, USA) and anti-α-smooth muscle actin (aSMA) (1A4 clone, Sigma Aldrich Química, Madrid, Spain) monoclonal antibodies, and isotype specific Alexa 488 and Alexa 594 labeled secondary antibodies (Molecular Probes, Invitrogen, Eugene, OR). Sections were counterstained with 4′,6-diamidino-2-phenylindole (DAPI). Immunoperoxidase staining of T-cells, B-cells, macrophages and PNAd+ high-endothelial venules (HEV) was performed and quantified as previously described [33]. Lymphoid neogenesis was defined by the presence of grade 2–3 T/B cell aggregates containing HEV as described [33].

For lymphatic vessels, immunoperoxidase labeling was performed with anti-podoplanin mAb (D2/40 clone, Dako) and avidin-biotin immunoperoxidase secondary reagents (Vector Laboratories, Burlingame, CA, USA), and developed by diaminobenzidine chromogen. Double lymphatic and CD31 labeling was performed by simultaneous podoplanin immunoperoxidase and CD31 immunofluorescent detection as indicated above.

The whole area of each tissue was photographed and digitalized using a Spot RT CCD camera and Spot 4.0.4 software (Diagnostic Instruments, Sterling Heights, Michigan) on a Zeiss Axioplan-2 fluorescence microscope (Zeiss, Jena, Germany). The number of blood vessels per area was determined by two independent observers blind to the origin and characteristics of each biopsy. Interobserver correlation coefficient for CD31+/aSMA- number of vessels was r = 0.75 (p<0.0001, Spearman's test). The proportion of labeled/unlabeled synovial tissue area was also analyzed in digitalized images using ImageJ software (http://rsb.info.nih.gov/ij).

### Statistical Analyses

For cross-sectional analyses, quantitative variables were compared by Mann Whitney U test or ANOVA (Kruskall Wallys test) where appropriate. Correlation between different numerical variables was analyzed by Spearman's test. Changes in quantitative variables before and after anti-TNF therapy were tested with Wilcoxon's signed rank test for paired data. Bonferroni correction was applied for the correction of multiple testing.

## Results

### Vascularity and Immature aSMA-Negative Blood Vessels in RA Synovial Tissues

**Figure 1:**
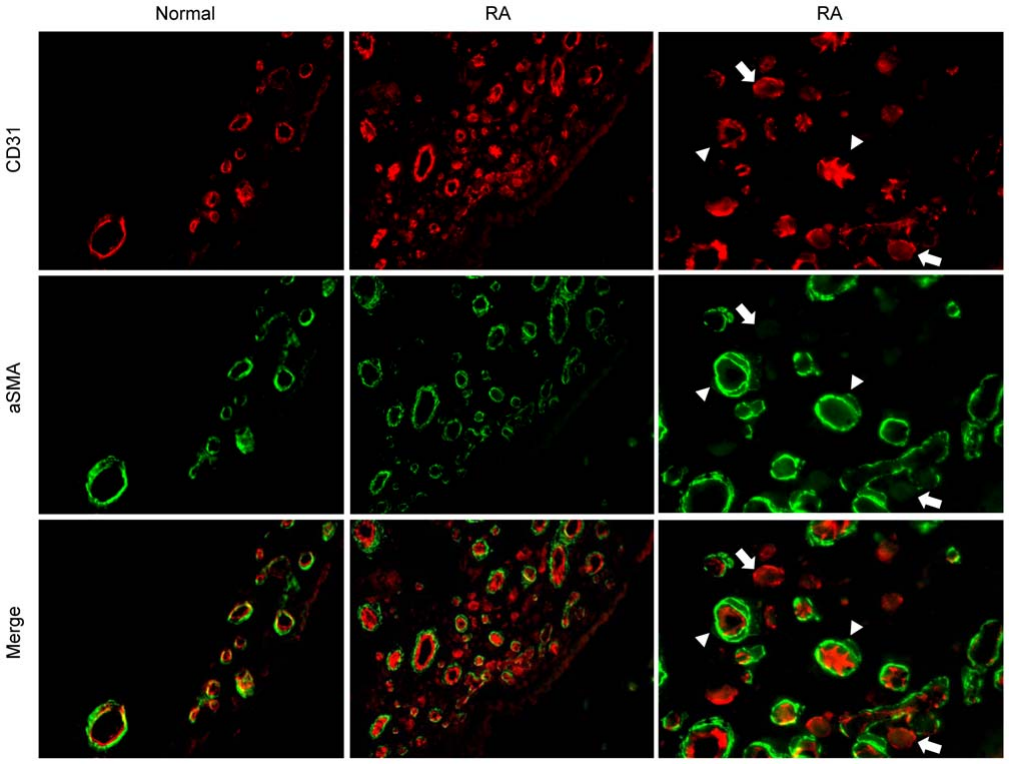
Detection of immature or mature blood vessels in RA synovial tissues. Double immunoflurescent labeling of endothelium (CD31, red fluorescence) and pericytes/smooth muscle cells (aSMA, green fluorescence) in normal and RA synovial tissue is shown. Original magnification ×400. Right panels show the same area as in middle panels with higher magnification. Mature CD31+ vessels covered by aSMA+ periendothelial cells are marked by arrows, and immature CD31+ vessels lacking aSMA+ mural cells by arrow heads.

By double labelling of endothelium (CD31) and pericyte/smooth muscle cells (aSMA) immature, CD31-positive vessels lacking aSMA-positive periendothelial cells, and mature CD31-positive vessels covered by aSMA-positive mural cells were identified in RA synovial tissues (Figure 1). Most RA tissues (66/82) contained a variable number of immature CD31+/aSMA- vessels, whereas they were only present in a small proportion of OA tissues at a significantly lower density, and were not identified in normal synovial tissues (Table 1). RA immature vessels were predominantly small size vessels, preferentially located in sublining areas containing abundant inflammatory infiltrates. Complete or partial concordance in the presence of immature vessels in different areas of the same joint was 30% and 53% respectively, whereas in 17% of the cases, only one area contained immature vessels.

**Table 1:** Mature and Immature Vessels in RA, OA or Normal Synovial Tissues.

	RA n = 82	OA n = 14	Normal n = 4	p-value*
CD31+/aSMA+ Vessels/mm^2^	294±95	74±28	94±44	<0.0001
CD31+/aSMA– Vessels/mm^2^	26±27	0.6±1.2	0±0	<0.0001
Total Vessels/mm^2^	319±98	74.5±28	94±44	<0.0001
Proportion of tissues with CD31+/aSMA– vessels (%)	66/82(80%)	3/14(21%)	0/4(0%)	<0.0001

CD31+/aSMA+: Mature vessels; CD31+/aSMA–: Immature vessels; Total vessels represents the sum of both mature and immature vessels.

(*) RA versus OA.

The fractional CD31-positive and aSMA-positive areas and the total number of mature vessels per area were also significantly increased in RA tissues compared to OA and normal tissues (Table 1). Although both determinations are not equivalent, since labelled area depends on number and size of vessels, statistically significant correlation was confirmed between manually acquired quantitative data on CD31- or aSMA-positive vessels per area and the fractional CD31- or aSMA-positive area evaluated by digital image analysis (r = 0.35, p = 0,001 and r = 0.31, p = 0.002 respectively).

**Figure 2:**
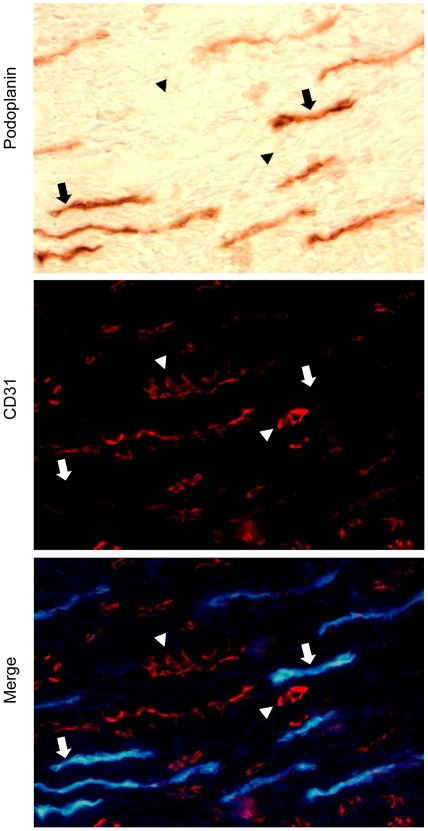
Double labeling of lymphatic and CD31-positive vessels in RA synovial tissues. Lymphatic vessels were detected by immunoperoxidase (brown immunostaining) detection of podoplanin and double immunofluorescent labeling (red fluorescence) of CD31. The same field was photographed by light or fluorescent microscopy to show the position of CD31+ (arrowheads) and podoplanin+ vessels (arrows). Light microscopy image was inverted and merged with CD31 fluorescent image of the same field to show the relative position of podoplanin (blue) and CD31 (red) labeling.

Weak CD31 labelling has occasionally been found in lymphatic vessels of different tissues [34]. Although erythrocytes could be observed in some immature vessels lumen by light phase contrast microscopy (data not shown), to formally exclude that increased lymphatics in RA could explain the presence of CD31 vessels lacking aSMA-positive periendothelial cells, we performed double CD31 and lymphatic (podoplanin) immunolabelling. Podoplanin was detected by peroxidase immunohistochemistry due to lower sensitivity of immunofluorescent labelling. Podoplanin immunoperoxidase and CD31 immunofluorescent labelling were mutually exclusive, therefore confirming that in RA synovial tissues, CD31+/aSMA- were immature blood vessels (Figure 2).

### Clinicopathological Correlations of Immature Blood Vessels in RA Synovial Tissues

**Figure 3:**
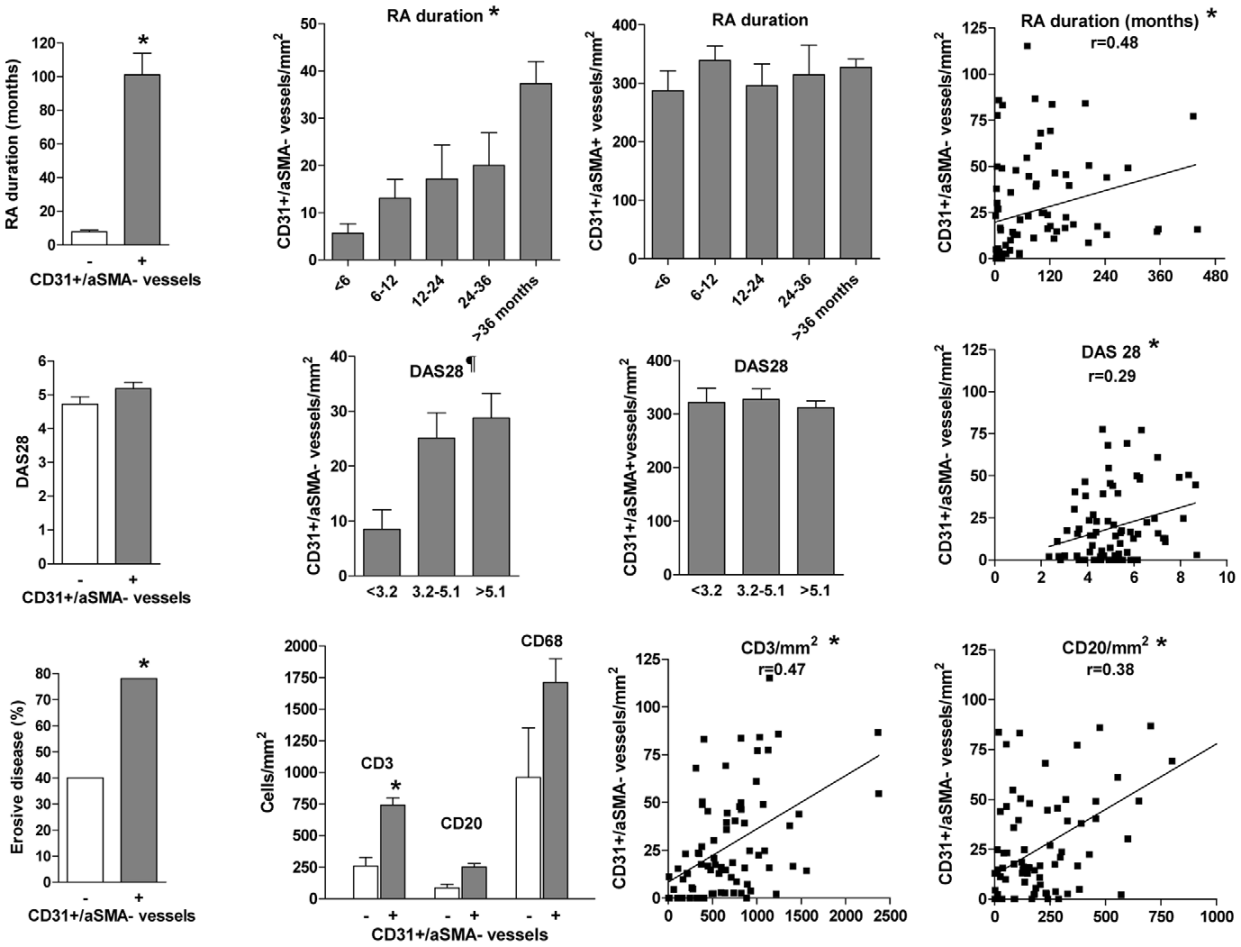
Clinicopathological correlations of immature blood vessels in RA synovial tissues. Disease duration, DAS28 score, erosive disease, and synovial tissue infiltration by CD3, CD20 or CD68 cells is shown in groups with (+) or without (−) immature vessels as indicated. Density of mature or immature vessels in patients stratified by disease duration and levels of activity (low: DAS28<3.2, moderate 3.2–5.1, or high>5.1). Spearman's correlation coefficients between immature vessels density and disease duration, DAS28, CD3 or CD20 infiltration are shown. (*) p<0.05 (see text). ¶ p = 0.04 (Kruskall Wallys test and post hoc Dunns test (low versus moderate or high activity groups).

Our RA patients represented a non-selected cross-sectional sample, heterogeneous in terms of disease duration, and demographic, clinical and analytical characteristics. We analysed whether selected characteristics of the disease, particularly disease duration and several markers of activity or severity, were correlated with the presence or abundance of immature vessels in synovial tissue (Table 2). The presence of immature vessels was significantly associated to a significantly longer disease duration (101±104 versus 7.8±3.6 months; p<0.0001; Table 2; Figure 3). The density of immature blood vessels was also significantly and positively correlated with the duration of the disease (p = 0.003; Figure 3). In contrast, the mature vascular density did not correlate with disease duration (Figure 3). The different density of immature and mature vessels stratified by different disease duration segments is shown in Figure 3.

**Table 2:** Clinicopathological data stratified by the presence of Immature Vessels (IV).

	All patients n = 82	IV- n = 16 (20%)	IV+ n = 66 (80%)	p-value*
Age (years)	58±13	53±10	59±10	NS
Female (%)	68%	69%	68%	NS
RA duration (months)	83±100	7.8±3.6	101±104	<0.0001
DAS28	5.1±1.4	4.7±0.8	5.2±1.5	NS
CRP (mg/dl)	4.02±3.43	3.08±2.92	4.25±3.50	NS
Erosive disease (%)	67%	40%	78%	0.0039
Auto-antibodies positive† (%)	71%	65%	72%	NS
CD3+ T-cells/mm^2^	667±466	258±239	742±460	0.0002
CD20+ B-cells/mm^2^	226±205	87±98	251±211	NS¶
CD68+ cells/mm^2^	1643±1326	990±1037	1729±1336	NS
LN (%)	48%	29%	52.4%	NS¶
Mature Vessels/mm^2^	294±95	279±111	298±92	NS

Data represent baseline data recorded at the time of biopsy. IV: immature CD31+/aSMA– vessels. LN: lymphoid neogenesis; DAS28: disease activity score; CRP: C-reactive protein; NS: Non-significant.

(*)IV- versus IV+ groups.

(†)RF or ACPA auto-antibodies.

(¶)p<0.05 but NS after correction for multiple testing.

Disease activity at biopsy as evaluated by DAS28 score was slightly higher in patients with synovial immature vessels (5.2±1.5 versus 4.7±0.8; Table 2 and Figure 3) but the difference did not reach statistical significance. The density of immature vessels was significantly lower in low versus moderate and high disease activity groups as shown in Figure 3 (p = 0.04). The density of immature blood vessels was also significantly and positively correlated with the DAS28 score (p = 0.009; Figure 3). Mature vascular density was not correlated with disease activity.

The presence of erosive disease at the time of biopsy was significantly higher in the group of patients with synovial immature vessels (78% versus 40%; p = 0.0039; Table 2). Stratification by sex, age, presence or absence of RF or ACPA auto-antibodies, did not show differences in the presence or density of immature vessels nor in mature vascular density in the different groups (Table 2).

Synovial inflammation was also quantified as the density of cellular infiltration by macrophages, T-cells, B-cells, or their organization into lymphoid aggregates characterized as lymphoid neogenesis (LN) as previously described (33). Correlation between these parameters and the presence or density of immature vessels was analysed. Tissues containing immature vessels contained a higher density of T and B-cells and macrophages (Table 2; Figure 3). A significant correlation between the density of T-cells (p<0.0001) but not sublining macrophage infiltration and the density of immature vessels was found (Figure 3). Density of B-cells and the presence of LN structures were higher in tissues with immature vessels but after correction for multiple testing the difference was non-significant. No significant correlation between mature vascular density and cell infiltration by any cell type or LN structures was found.

### Effects of Anti-TNF Therapy on Mature and Immature Blood Vessels

To evaluate whether the structure or abundance of immature vessels was modified by therapy and whether these changes correlate with changes in the clinical course of the disease induced by therapy, we analysed a subgroup of 25 patients rebiopsied after anti-TNFα therapy. Clinical and synovial cellular changes in response to therapy in this group of patients are shown in Table 3.

**Table 3:** Clinicopathological changes in patients after anti-TNFα therapy.

	Basal Biopsy	Post Anti-TNF Biopsy	p-value	Δ Change Non-responders*	Δ Change Responders
DAS28	6.0±1.4	3.9±1.9	<0.0001	−0.5±0.7	3.2±1.8
CRP mg/dl	4.8±3.5	1.6±2.0	0.0004	1.0±4.1	4.2±3.6
CD31+/aSMA– vessels/mm^2^	52±31	31±27	0.017	−0.4±13	12±16
CD31 area (%)	3.1±1.6	2.5±1.4	0.03	−0.2±2.1	0.9±1.0
CD31+/aSMA+ Vessels/mm^2^	276±82	321±110	NS	30±25	8±59
aSMA+ area (%)	3.68±1.6	3.60±1.4	NS	−0.1±1.9	0.1±1.7
CD3/mm^2^	823±88	478±83	0.0064	202±398	400±698
CD20/mm^2^	322±59	220±54	NS	219±245	56±411
CD68/mm^2^	324±38	192±31	0.0091	53±180	163±195

CD31+/aSMA–: immature vessels. CD31+/aSMA+: mature vessels. p-value of basal versus post-anti-TNF values.

(*)Absolute decrease from basal values in patients achieving moderate or good EULAR response (responders) and non responders to anti-TNF.

After therapy, a significant improvement in DAS28 scores was observed, as well as a significant decrease in T-cell and macrophage cell infiltration, and a non-significant decrease in B-cell infiltration (Table 3). Seven patients had not responded, and 6 and 12 had obtained moderate and good EULAR responses to anti-TNF-α therapy at the time of the second biopsy [35].

**Figure 4:**
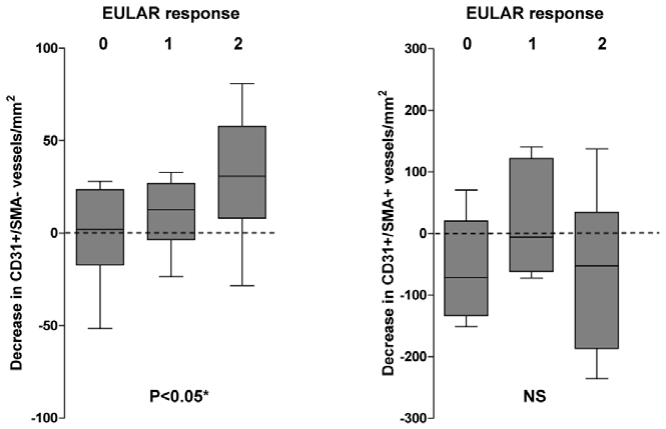
Variation in the density of immature vessels stratified by the levels of response to anti-TNF-α therapy. Decrease in immature (left graphics) or mature (right graphics) vessels density between the first and second biopsy after anti-TNF-α therapy is shown stratified by EULAR responses: 0 = : No response; 1: Moderate response; 2: Good response. (*) Kruskall Wallys test and post hoc Dunns test (non-responders versus good responders).

A statistically significant decrease in the number of CD31+/aSMA- immature vessels was observed in biopsies obtained after anti-TNF-α therapy (Table 3). In contrast, the number of mature CD31+/aSMA+ vessels per mm^2^ was not significantly modified after therapy (Table 3). Consistently, the fractional CD31 area was significantly decreased after therapy whereas the aSMA area was not modified (Table 3). The relative decrease in immature vessels density was higher in patients obtaining better EULAR therapeutic responses (p = 0.01; Figure 4). Clinical and pathological changes in responders and non-responders are also shown in Table 3.

## Discussion

The presence of immature blood vessels is a phenomenon previously associated to cancer tissues or developmental processes where active angiogenesis takes place [21], [36]. An imbalance between endothelial cell tube formation and the parallel development of pericytes has been mechanistically linked to VEGF-induced angiogenesis [23]. In RA, a severely hypoxic environment and possibly cytokines, induce Hif (hypoxia inducible factor) mediated transcriptional activation of VEGF and many other pro-angiogenic genes [10], [26], [27], [37]. Excessive expression of VEGF in chronically inflamed RA synovial tissue is therefore one of the key factors explaining increased angiogenesis in the synovial membrane [5]–[9]. Consistently, we found abundant immature blood vessels in the inflammatory RA tissue which represents the first description of this vascular abnormality in a human chronic inflammatory disease. Scanty immature vessels could also be detected in a few OA but not in normal synovial tissues. This suggests that active angiogenesis and the presence of immature vessels is not a disease-specific process but it is rather associated to the severity of inflammation. In OA, a less intense inflammatory process and active vascular remodelling are also variably present [2]–[4]. In a previous study we found that increased VEGF expression also characterizes OA synovial fibroblasts [38]. In RA, immature vessels seem to appear relatively early in the disease but as disease progresses their density increases, being maximal in long-standing, active, and erosive disease groups. The observed correlation between lymphocyte infiltration and immature vessels formation points to a possible link between both processes.

Among patients rebiopsied after anti-TNF-α therapy, immature vessels depletion was preferentially observed in those patients achieving good therapeutic responses. The important increase in mature vessels density observed in RA tissues seems present from the earliest phases and less susceptible to change. After anti-TNF-α therapy, the observed decrease in immature vessels was not paralleled by a reduction in mature vasculature. Consistently, only CD31 but not aSMA labelled area was decreased by anti- TNF-α therapy. These observations together with previous observations on the effect of therapy on local or systemic angiogenesis markers suggests that effective therapy halts active angiogenesis but has little effect on the expanded mature vascular bed [10], [28], [29].

In the most refractory patients, immature vascular development seems insufficiently targeted by anti-TNF-α therapies. Persistently enhanced vascularity after improvement of clinical inflammation can be a factor of chronicity and it has been associated to further progression of joint damage [30]–[32]. Whether the persistent vascular signal observed by imaging studies corresponds to resistant immature or to higher mature vascularity is not known. Parallel imaging and histological studies are needed to evaluate the contribution of persistent immature/mature vessels to disease progression and may illustrate the specific pathogenetic contribution of immature vessels.

In cancer tissues, specific anti-angiogenic anti-VEGF therapy has been found to induce selective changes in the immature vascular bed, a process called vascular normalization, where immature vessels selectively disappear [21], [24]. This is consistent with the different sensitivity to VEGF depletion of immature and mature vessels. Whereas VEGF is required to sustain newly formed vessels, this factor is dispensable for the mature vascular network [21]. Our data suggest that upon indirect VEGF down-regulation by anti-TNF-α therapy in RA [10]–[12], blood vessels normalization rather than global vascular reduction occurs, and suggests that VEGF antagonists might not be active on the largest fraction of the expanded synovial vascularity. In an animal model of airway inflammatory angiogenesis, VEGF independent angiogenic effects of TNF-α have also been identified, suggesting that alternative mediators may also be linked to the down-regulation of inflammatory angiogenesis induced by TNF-α blocking [39].

Although immature vessels depletion occurred preferentially in patients responding to anti-TNF-α therapy, the pleiotropic effects of this intervention do not permit to speculate on the role of immature vessels depletion in such response. In cancer, immature vessels are associated to increased permeability and high interstitial fluid pressure, decreasing the effective perfusion of the tissue and drug access, and modifying inflammatory cell infiltration [20]–[22], [25].

The role of immature vessels in inflammation has only been explored in a murine model of Mycoplasma induced airway inflammation [40]. In this model, enforced vascular immaturity by ephrinA2 deletion was directly linked to greater leukocyte infiltration and higher expression of inflammatory cytokines upon inflammatory challenge. Further studies on the specific contribution of immature blood vessels to RA pathogenesis are needed to understand the potential of more specific anti-angiogenic interventions for the therapy of RA.
